# Leukocyte telomere length and sarcopenia-related traits: A bidirectional Mendelian randomization study

**DOI:** 10.1371/journal.pone.0296063

**Published:** 2024-01-02

**Authors:** Dingkun Wang, Chenhao Li, Xinwen Zhang, Yihao Li, Junhua He, Xiaoming Guo

**Affiliations:** 1 Department of Neurosurgery, Tongde Hospital of Zhejiang Province, Hangzhou, China; 2 Department of Neurosurgery, The Second Affiliated Hospital, Zhejiang University School of Medicine, Hangzhou, China; Tulane University Health Sciences Center, UNITED STATES

## Abstract

Accumulating evidence indicated that leukocyte telomere length (LTL) was related to sarcopenia. However, it is still not clear whether the association of changes in LTL with sarcopenia is likely to be causal, or could be explained by reverse causality. Thus, we carried on bidirectional Mendelian randomization (MR) and multivariable MR analyses to identify the causal relationship between LTL and sarcopenia-related traits. Summary-level data and independent variants used as instruments came from large genome-wide association studies of LTL (472,174 participants), appendicular lean mass (450,243 participants), low grip strength (256,523 participants), and walking pace (450,967 participants). We identified suggestive association of longer LTL with larger appendicular lean mass [odds ratio (OR) = 1.053; 95% confidence interval (CI), 1.009–1.099; P = 0.018], and causal association of longer LTL with a lower risk of low grip strength (OR = 0.915; 95% CI, 0.860–0.974; P = 0.005). In the reverse MR analysis, we also observed a positive causal association between walking pace and LTL (OR = 1.252; 95% CI, 1.121–1.397; P < 0.001). Similar results can be repeated in sensitivity analyses. While in the multivariable MR analysis, the estimate of the impact of walking pace on LTL underwent a transformation after adjusting for T2DM (OR = 1.141; 95%CI: 0.989–1.317; P = 0.070). The current MR analysis supported a causal relationship between shorter telomere length and both low muscle mass and strength. Additionally, walking pace may affect LTL through T2DM.

## Introduction

Telomeres are nucleoprotein structures comprising thousands of repeats of the non-coding sequence TTAGGG, which are crucial in maintaining chromosomal integrity and the accurate replication of the entire human genome [1, 2]. With each round of cell division, telomeres shorten by approximately 50–100 base pairs in length due to the inability of deoxyribonucleic acid (DNA) polymerase enzyme to fully replicate the terminal regions of linear DNA [3, 4]. When telomeres reach a critically short length, they lose their normal function and trigger DNA damage responses that can lead to cellular apoptosis or senescence [5–7]. Telomere dysfunction has been implicated in a range of age-related diseases, including cancer, cardiovascular disease, diabetes mellitus, and other common adult conditions [8–12].

Sarcopenia is a geriatric disorder marked by a gradual and progressive decrease in skeletal muscle strength, loss of skeletal muscle mass, and decline in physical performance. And severe sarcopenia was defined as all the three conditions aforementioned being present [13]. Sarcopenia is prevalent in older people and significantly elevates the risks of disability, falls and falls-related injuries, limitation of independence, and mortality [14, 15]. Although numerous studies have investigated the association between sarcopenia and telomere length, the results remained controversial [16–22]. Observational associations may be confounded by a number of factors, including sex and race, smoking, paternal age at birth, psychological stress, and other psychosocial, environmental, and behavioral factors [23, 24]. In addition, despite the fact that telomere length is widely considered as an ageing biomarker, it was also unclear whether changes in telomere length were the causes or consequences of sarcopenia. Therefore, it is necessary to clarify the direction of association between telomere length and sarcopenia.

According to a meta-analysis of six independent cohort studies, telomere length is largely heritable and is affected by genetic factors [25]. And telomere length is generally estimated by leukocyte telomere length (LTL), which is readily to obtain from blood and strongly correlated with telomere length in other tissues [26]. Although the genetic architecture underlying sarcopenia is not fully elucidated, current GWASs provide evidence that muscle phenotypes and the risk of sarcopenia are heritable and regulated by genetic factors [27–30]. Mendelian randomization (MR) is a powerful genetic epidemiologic approach by utilizing genetic variants associated with exposures, which can avoid potential methodological limitations of observational studies, such as confounding and reverse causation bias [31]. Given that both LTL and sarcopenia are influenced by genetic factors, using MR analysis to enhance our understanding of the relationship is promising.

## Materials and methods

### Study design

**Fig 1:**
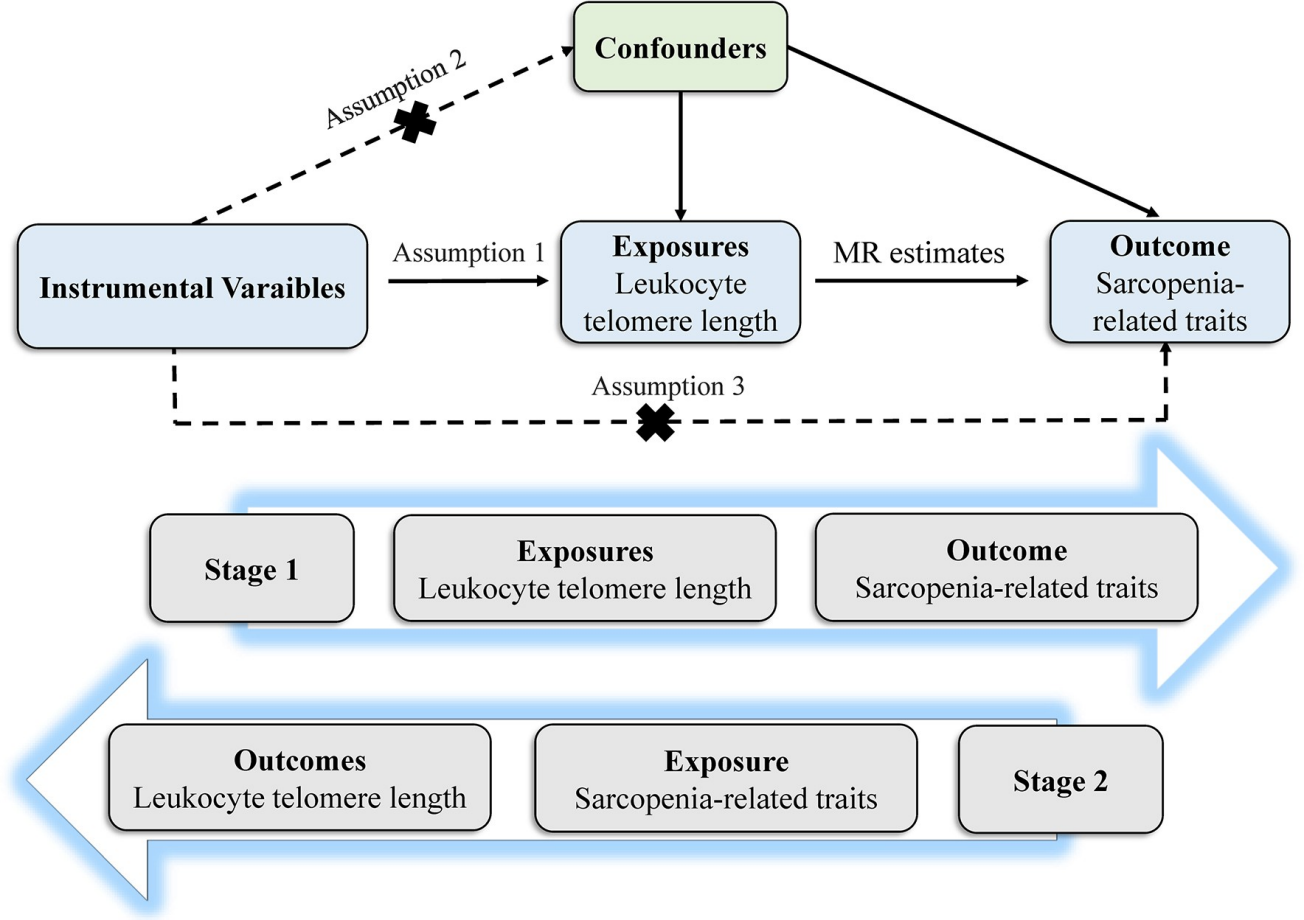
Principles of the Mendelian randomization study for leukocyte telomere length and sarcopenia-related traits.

This bidirectional MR study relies on three predominant assumptions (Fig 1): 1) The selected instrumental variables (IVs) should be robustly associated with exposures; 2) The selected IVs should not be associated with potential confounders; 3) The IVs should impact the outcomes through exposures directly without any other pathways. We performed this bidirectional MR study to investigate whether LTL was associated with sarcopenia. In the forward MR analyses, LTL was considered as the exposure and sarcopenia-related traits were considered as the outcomes, whereas in the second MR analyses, the roles were reversed with sarcopenia-related traits as the exposures and LTL as the outcome.

### Instrumental variables

Appropriate IVs for MR analyses were obtained from several different genome wide association study (GWAS) summary results. Single nucleotide polymorphisms (SNPs) strongly associated with exposures were selected as IVs (P < 5.0×10^−8^ or P < 5.0×10^−9^) All selected IVs were clumped for independence according to the Europeans data from the 1000 Genomes Project (r^2^ < 0.1; region size, 3000 kb). If the SNPs included in these datasets were not present in the outcome datasets, we obtained proxy SNPs (with r^2^ > 0.8) as replacements online (ldlink.nci.nih.gov/). Palindromic SNPs were removed in the MR analyses when harmonizing the directions of SNP effects on exposures and outcomes. We also assessed the F‐statistics to evaluate the instrument strength. F‐statistics < 10 indicated weak instrument bias [32].

### Data sources

Summary results associated with LTL were obtained from a GWAS, which were based on 472,174 predominantly healthy participants in the UK Biobank [33]. LTL was measured using a well-validated quantitative polymerase chain reaction assay and was expressed as the ratio of the telomere repeat number to a single-copy gene. In total, 197 significant SNPs were reported to be associated with LTL at a level of genome-wide significance, which could explain 4.54% of the variance in LTL.

Regarding the sarcopenia-related traits, we selected appendicular lean mass (ALM) as a measure of muscle mass, low grip strength as a measure of upper limb muscle strength and walking pace as a measure of physical performance. ALM is mainly affected by skeletal muscle and is commonly used in the EWGSOP-2 due to its high predictive power for sarcopenia [13]. We used a GWAS of ALM from the UK Biobank (n = 450,243), in which ALM was measured using bioelectrical impedance analysis (BIA) for fat-free mass at the arms and legs [28]. We obtained 1059 SNPs associated with ALM (P < 5.0×10^−9^) for the analyses, which explained 15.5% of the variance. As for muscle strength, the data of low hand grip strength was extracted from a genome-wide meta-analysis of 22 independent cohorts, which comprised 256,523 individuals aged > 60 years [29]. Low grip strength was defined as grip strength <30 kg (male) or <20 kg (female) according to EWGSOP [34]. The estimate of the SNP-based heritability for low grip strength was 4.4%. For walking pace, the data (n = 450,967) was obtained from the UK Biobank by answering the question “How would you describe your usual walking speed”. The options included “slow” (less than three miles per hour), “steady/average” (between 3 to 4 miles per hour), and “fast” (more than four miles per hour) [30]. The SNP-based heritability estimate for walking pace was 13.2%. The variants associated with LTL and sarcopenia related traits were all adjusted by age, sex, genotype array and the principal components.

In the studies included, all participants provided written informed consent, and the involved sites obtained approval from local research ethics committees or Institutional Review Boards.

### Statistical analysis

The random-effects inverse-variance weighted (IVW) approach was adopted as the main analysis to evaluate the bidirectional relationship between LTL and sarcopenia-related traits [35]. For sensitivity analyses, we used several methods to identify potential pleiotropy. The Cochran’s Q test was performed to assess the heterogeneity among included IVs [35]. The weighted median method was employed because it allows for less than 50% of the genetic variants to be considered invalid IVs [36]. MR-Egger method was conducted to adjust directional pleiotropic bias [37]. We additionally performed the MR Pleiotropy Residual Sum and Outlier (MR-PRESSO) method to identify horizontal pleiotropy [38]. Upon identification of pleiotropic outlier instruments, a subsequent IVW analysis would be conducted following the exclusion of these outlier instruments. Furthermore, multivariable MR analyses were applied to adjust for estimates independent of the effects of some important metabolic syndrome traits [39]. We obtained public summary statistics for body mass index (BMI), low-density lipoprotein cholesterol (LDL-c), and type 2 diabetes mellitus (T2DM) [40–42].

In our analyses, the tests were two sided and the significance threshold was set to P ≤ 0.008 due to Bonferroni correction. The P values between 0.05 and 0.008 was defined as suggestive of potential association between exposures and outcomes. All analyses were conducted by utilizing packages of MendelianRandomization, TwoSampleMR, and MR-PRESSO in R version 4.1.3.

## Results

The characteristics of GWASs included in the MR analyses were shown in S1 Table. The summary information of SNPs on the LTL and sarcopenia-related traits was shown in S2 and S3 Tables.

### Impact of LTL on sarcopenia-related traits

**Fig 2:**
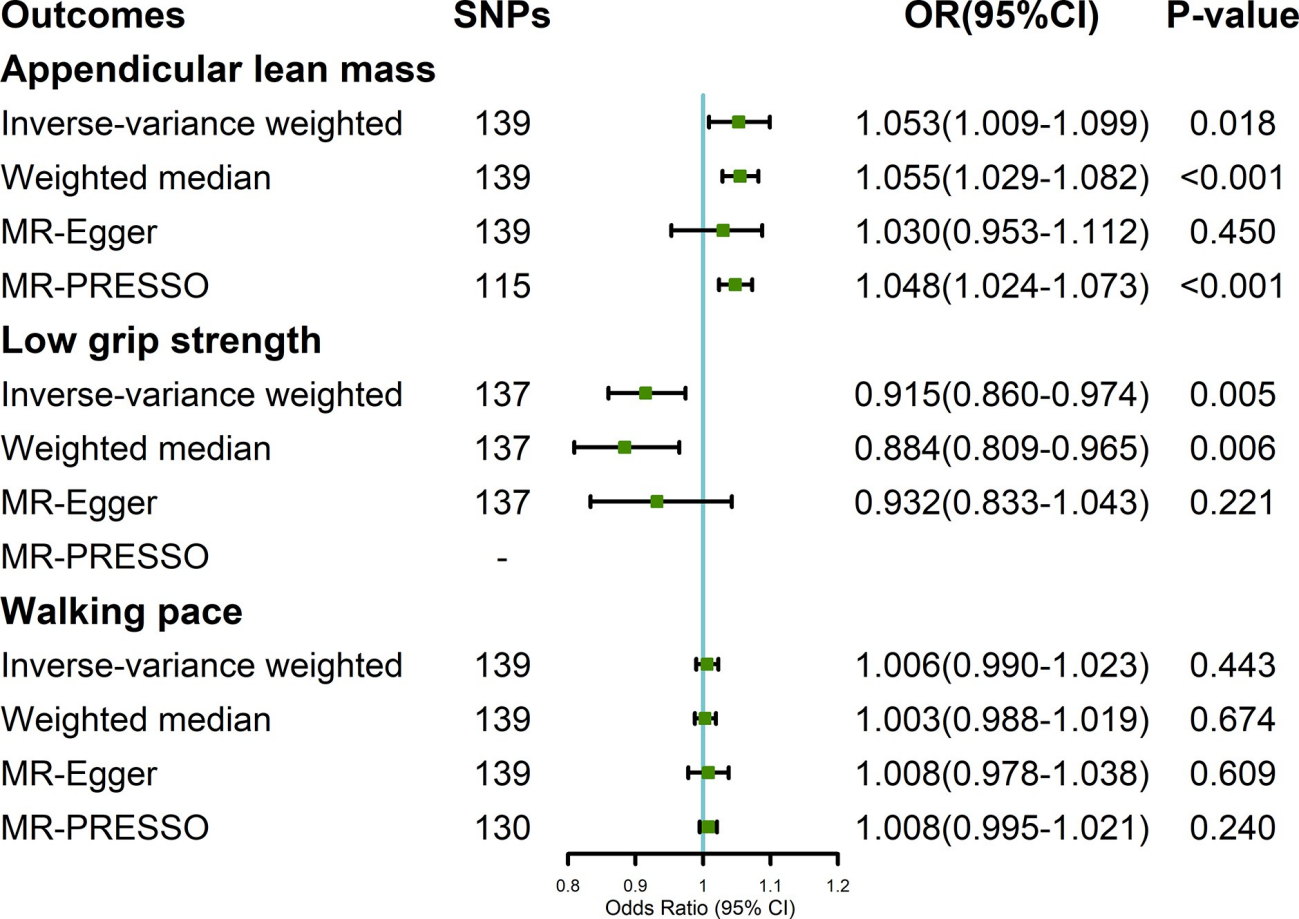
The effect of leukocyte telomere length on sarcopenia-related traits.

The average F-statistics of LTL was 130.38, which meant that the included IVs were powerful enough (F-statistics > 10) to minimize potential bias. In the random-effect IVW estimates, longer LTL was potentially associated with larger ALM [odds ratio (OR) = 1.053; 95% confidence interval (CI), 1.009–1.099; P = 0.018; Fig 2 [The sensitivity analyses indicated consistent results. The result of MR pleiotropy test indicated 24 potential pleiotropic IVs for ALM (S2 Table). After removing these outliers, the IVW estimate showed that LTL had a significant causal impact on ALM. As for muscle strength, the IVW estimates indicated that longer LTL was causally associated with a reduced risk of low grip strength (OR = 0.915; 95% CI, 0.860–0.974; P = 0.005). The sensitivity analyses also showed similar results and no outlier was found for low grip strength in MR pleiotropy test. Moreover, there was no evidence suggesting a causal effect of LTL on walking pace (OR = 1.006; 95% CI, 0.990–1.023; P = 0.443). There was no evidence of horizontal pleiotropy found in stage 1 (S4 Table).

### Impact of sarcopenia-related traits on LTL

**Fig 3:**
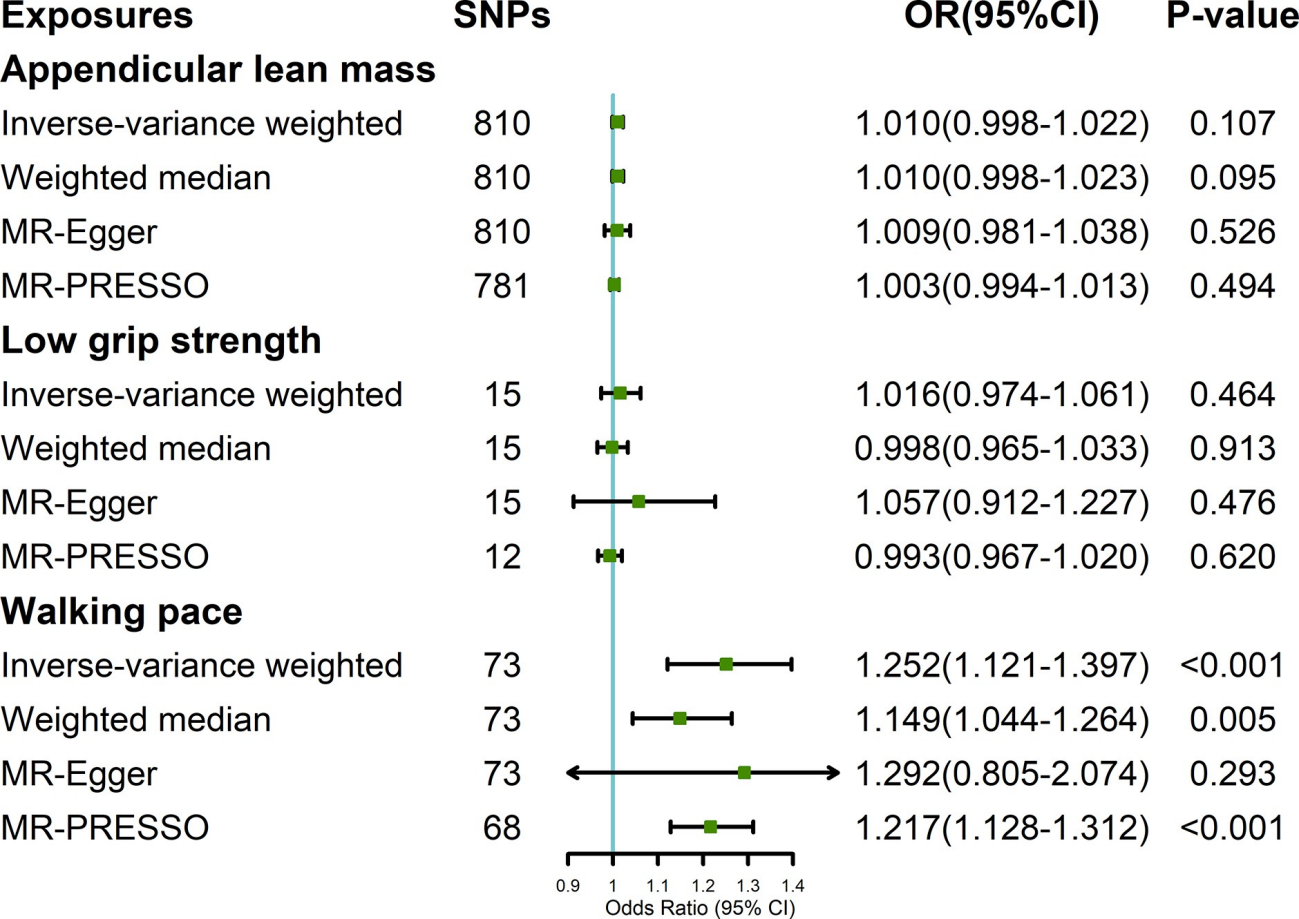
The effect of sarcopenia-related traits on leukocyte telomere length.

In the reverse stage, the average F statistics of ALM, low grip strength and walking pace were 78.78, 283.99, and 14.01 respectively (S3 Table). As shown in Fig 3, MR analysis by IVW method revealed that ALM had no causal effect on LTL (OR = 1.010; 95% CI, 0.998–1.022; P = 0.107). Additionally, no evidence was shown supporting the causal relationship between lower grip strength and LTL (OR = 1.016; 95% CI, 0.974–1.061; P = 0.464). The influence of walking pace on LTL was also studied. A positive causal association was found between walking pace and LTL (OR = 1.252; 95% CI, 1.121–1.397; P < 0.001). Similar effect estimates could be observed in WM and MR- Egger methods. The MR-PRESSO identified 5 potential SNP outliers and the result remained consistent after removing the outliers (OR = 1.217; 95% CI, 1.128–1.312; P < 0.001). There was no indication of horizontal pleiotropy detected in stage 2 (S4 Table).

### Multivariable MR adjusted for BMI, LDL-c and T2DM

**Fig 4:**
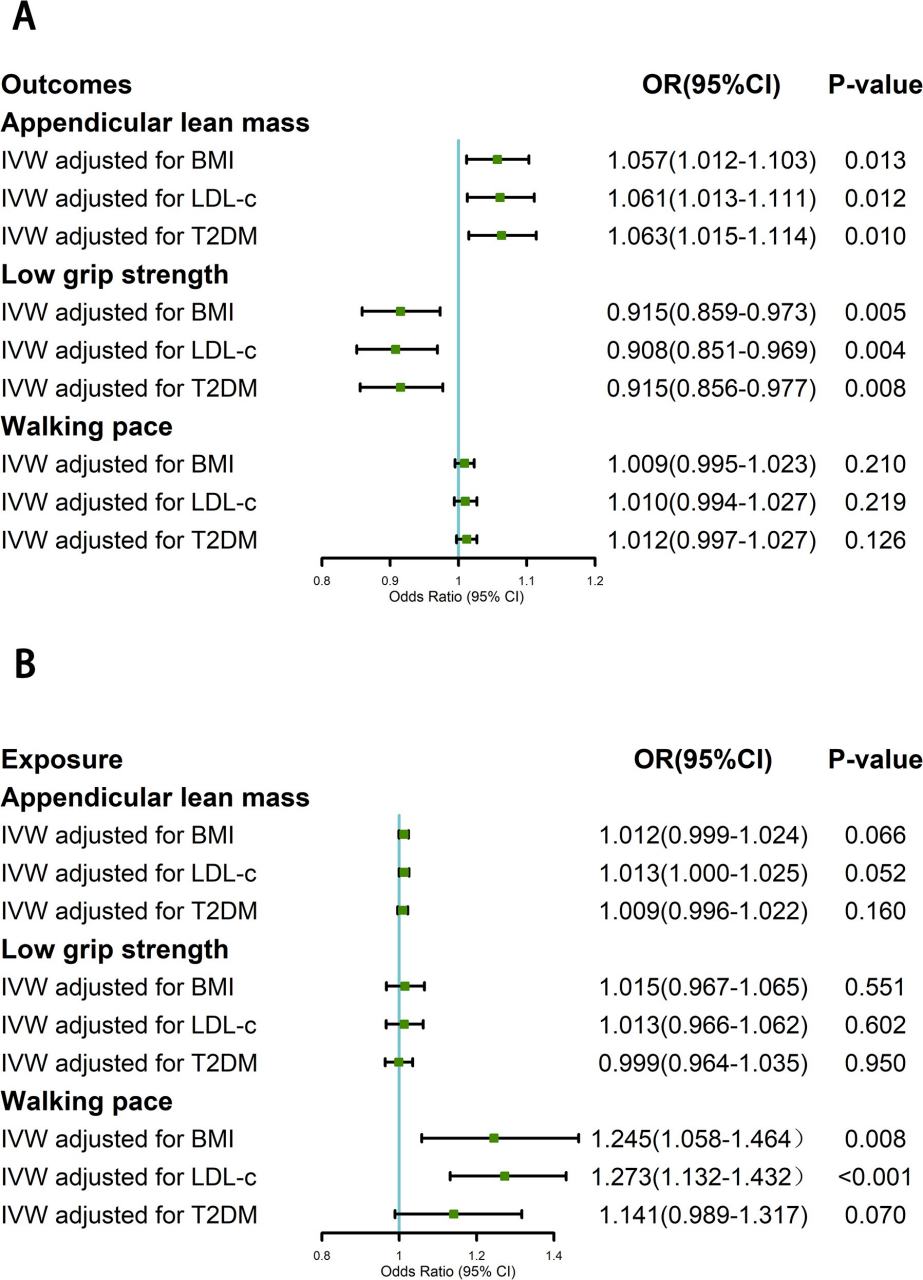
Multivariable MR analyses adjusting for BMI, LDL-c, and T2DM in two directions. BMI: body mass index, LDL-c: low-density lipoprotein cholesterol, T2DM: type 2 diabetes mellitus.

In the part of forward direction, the results remained basically consistent with the outcomes observed in the univariable MR analyses (Fig 4). But in the results of reverse direction, the impact of walking pace on LTL underwent a transformation after adjusting for T2DM (OR = 1.141; 95%CI: 0.989–1.317; P = 0.070).

## Discussion

We conducted current MR study to assess the bidirectional causal association between LTL and sarcopenia. In the forward MR analyses, longer LTL was suggestively associated with larger ALM, and shorter LTL was associated with higher risk of low hand grip strength. In the reverse MR analyses, faster walking pace was associated with longer LTL. Multivariable MR analyses suggested that T2DM appeared to mediate the effect of walking pace on LTL.

Overall, we reported a significantly bidirectional association between LTL and sarcopenia.

In a recent MR study investigating association between LTL and aging‐related outcomes and involving 261,000 participants from UK Biobank, the findings did not support a causal relationship between LTL and grip strength, as well as muscle mass [19]. One possible explanation for the distinct findings could be attributed to the limited SNPs they used, which might have resulted in an incomplete representation of the underlying genetic variations. We used the largest available GWAS data for LTL and sarcopenia-related traits, which could enhance statistical power to detect potential association between these traits. Overall, the findings of our MR analysis provided valuable and novel insights in the context of the current literatures, that is, shorter LTL is causally related to low muscle mass and strength, and declined physical performance causes shorter LTL, thus forming a closed loop.

According to current concepts around telomere dynamics, LTL is not simply a biomarker of ageing, but also has a complex relationship between specific health outcomes through selective evolutionary forces [43, 44]. The impact of shorter LTL on increased risk of sarcopenia could be explained by telomere attrition with each cycle of DNA replication, reduction in the number and replicative potential of satellite cells, and eventually leading to a decrease in skeletal muscle mass as well as decline in muscle function. Several prior studies have demonstrated that shorter LTL was found among individuals with sarcopenia compared to those without [16, 20–22]. However, as mentioned earlier, it still remains unclear of the direction of association between LTL and sarcopenia. A recent prospective study found a significant correlation between longer LTL at birth and higher lean mass in late infancy [45]. Another cross-sectional study investigated the relationship between skeletal muscle mass and LTL in the pediatric population (aged 6–11 years) and reached similar results [46]. These results to some extent supported the conclusion of our current MR analysis, as assuming shorter LTL to be a cause for muscle mass reduction may be more reasonable than assuming muscle mass reduction to be a cause for shorter LTL in infants and young children.

With increasing age, the impacts of decreased physical activity are prominently manifested in the legs, rather than the arms [47]. Walking speed is recommended by EWGSOP-2 as a convenient and effective measure to evaluate physical performance and predict outcomes related to sarcopenia [13]. Our current findings on the relationship between walking pace and LTL are generally consistent with previous studies in this area. A cross sectional study enrolled 1,476 older white and African American women and revealed that higher levels of moderate-to-vigorous physical activity and faster walking pace were associated with longer LTL [48]. Another randomized controlled trial further showed that 40 minutes of moderate-intensity aerobic exercise 3 to 5 times per week induced apparent telomere lengthening [49]. It should be emphasized that walking pace is an indicator of physical activity intensity rather than physical activity volume, which is closely associated with cardiorespiratory fitness [50]. And higher intensity of physical activity may stimulate anti-oxidant and anti-inflammatory responses, and upregulate mRNA expression of telomerase reverse transcriptase, which can decelerate the process of telomere attrition [51–54]. In addition, in the multivariable MR analyses of the impact of walking pace on LTL, the results changed from being significant to non-significant after adjusting for T2DM. This implies that walking pace may affect LTL through T2DM. Previous MR analyses suggested a bidirectional causal relationship between walking pace and T2DM [55]. Several epidemiological studies supported associations between shorter LTL and higher risk of T2DM [10, 56, 57]. Hence, our results may have implications for the exploration of underlying mechanisms in the relationship between walking pace and LTL.

One of the major strengths of this analysis is the well-powered GWAS data of LTL. Additionally, the use of bidirectional MR design permitted a comprehensive evaluation of the mutually causal relationship. However, there are several limitations that should be addressed. First, Sample overlap between the exposure and outcome populations can potentially bias study results. However, a recent study demonstrated that, except for MR-Egger, most two-sample MR methods can be safely and robustly employed for one-sample MR within large biobanks [58]. And this may contribute to the deviation of our MR-Egger estimates or wider CIs. Meanwhile, Second, the included GWAS of LTL is based on a one-time measurement, which may be limited by the nature of the cross-sectional study. Thus, further longitudinal studies are currently needed to evaluate the association between sarcopenia-related traits and repeated measurements of LTL over time among older adults in order to confirm and extend these findings. Third, the participants included in our study were of European descents, which limited the generalizability of our findings to other ancestries.

## Conclusion

We provided evidences that a bidirectional association between LTL and sarcopenia. Shorter LTL was causally related to decrease in muscle mass and decline in muscle strength. Conversely, walking pace may affect LTL through T2DM. Further research is warranted to confirm the bidirectional causal relationship, particularly among populations at heightened susceptibility to chronic diseases or age-related decline.

## Supporting information

S1 Checklist STROBE statement—checklist of items that should be included in reports of observational studies. (DOCX)

S1 Table Studies and datasets adopted in the MR analyses. (XLSX)

S2 Table Summary information of the single nucleotide polymorphisms used as instrumental variables for sarcopenia-related traits. (XLSX)

S3 Table Summary information of the single nucleotide polymorphisms used in reverse MR analyses. (XLSX)

S4 Table Results of potential pleiotropy and heterogeneity assessments in the bidirectional analyses. (XLSX)
